# An exploration of relaxation and meditation practices in the management of eating disorders

**DOI:** 10.1186/2050-2974-2-S1-P7

**Published:** 2014-11-24

**Authors:** Rebecca Meers, Geoff Lyons

**Affiliations:** 1 University of Wollongong, Wollongong, Australia

## 

Individuals with eating disorders often experience strong psychological distress, emotional regulation difficulties and engage in excessive physical exercise. These problems can also make it challenging for them to monitor their emotions, thoughts and bodily sensations. Recently, there has been increased interest in the use of Yoga in managing eating disorders. It is theorised that Yoga may improve body and psychological awareness and emotional regulation difficulties via mindfulness (the ability to nonjudgmentally attend to the present moment). However, there is also some evidence to suggest these practices, and Yoga in particular, with its emphasis on body-based awareness and self-reflective practices, are particularly challenging for this population. Empirical research is yet to explore the attitudes of such individuals to these management options. This presentation discusses preliminary data from a cross-sectional study exploring attitudes towards the use of Yoga and other mindfulness practices in people with eating disorders. This study will help develop much needed theory on the role of Yoga and mindfulness in the management of eating disorders as well as provide direction for psychologists, Yoga practitioners and medical staff who work with eating disorder clients.

